# Supplementary material for: Towards Automated Brain Aneurysm Detection in TOF-MRA: Open Data, Weak Labels, and Anatomical Knowledge
Source: Neuroinformatics. 2022 Aug 18;21(1):21–34. doi: 10.1007/s12021-022-09597-0 (PMC9931814; doi:10.1007/s12021-022-09597-0)
Supplement: Supplementary file 2 — Supplementary file2 (DOCX 183 KB) [file 12021_2022_9597_MOESM2_ESM.docx]

**Supplementary Material**

**Paper Title:**

Towards automated brain aneurysm detection in TOF-MRA: open data, weak labels, and anatomical knowledge

**Author information:**

Tommaso Di Noto^a [0000-0002-5161-055X]^, Guillaume Marie^a [0000-0002-2447-1056]^, Sebastien Tourbier^a [0000-0002-4441-899X]^, Yasser Alemán-Gómez^a,b [0000-0001-6067-8639]^, Oscar Esteban^a [0000-0001-8435-6191]^, Guillaume Saliou^a [0000-0003-3832-7976]^, Meritxell Bach Cuadra^a,c [0000-0003-2730-4285]^, Patric Hagmann^a [0000-0002-2854-6561]^, Jonas Richiardi^a [0000-0002-6975-5634]^

a. Department of Radiology, Lausanne University Hospital and University of Lausanne, Lausanne, Switzerland

b. Center for Psychiatric Neuroscience, Department of Psychiatry, Lausanne University Hospital and University of Lausanne, Lausanne, Switzerland

c. CIBM, Center for Biomedical Imaging, Lausanne, Switzerland

**Corresponding author:**

Tommaso Di Noto. **Email**: tommaso.di-noto@chuv.ch; **phone**: +41 779672516

Table 1: MR acquisition parameters of TOF-MRA scans of our study sample.

| **# scans** | **Vendor** | **Model** | **Field strength [T]** | **TR**  **[ms]** | **TE**  **[ms]** | **Voxel spacing**  **[**$\boldsymbol{mm}^{\boldsymbol{3}}$**]** |
| --- | --- | --- | --- | --- | --- | --- |
| 71 | Philips | Intera | 3.0 | 18.3 | 3.40 | 0.39 x 0.39 x 0.55 |
| 23 | Siemens  Healthineers | Aera | 1.5 | 24.0 | 7.0 | 0.35 x 0.35 x 0.5 |
| 49 | Siemens  Healthineers | Skyra | 3.0 | 21.0 | 3.43 | 0.27 x 0.27 x 0.5 |
| 34 | Siemens  Healthineers | Symphony | 1.5 | 39.0 | 5.02 | 0.39 x 0.39 x 1 |
| 42 | Siemens  Healthineers | TrioTim | 3.0 | 23.0 | 4.18 | 0.46 x 0.46 x 0.69 |
| 65 | Siemens  Healthineers | Verio | 3.0 | 22.0 | 3.95 | 0.46 x 0.46 x 0.7 |
| 12 | Siemens Healthineers | Prisma | 3.0 | 20.0 | 3.3 | 0.28 x 0.28 x 0.65 |

Table 2. List of anatomical landmark points and corresponding locations. ACOM = Anterior Communicating Artery; Pcom = Posterior communicating artery. MCA = Middle Cerebral Artery.

| Landmark point | Location |
| --- | --- |
| 1 | ACOM |
| 2 | Pcom right |
| 3 | Pcom left |
| 4 | Pericallosal proximal |
| 5 | Pericallosal distal |
| 6 | Carotid tip right |
| 7 | Carotid tip left |
| 8 | MCA right |
| 9 | MCA left |
| 10 | Basilar tip |
| 11 | Carotid extra right |
| 12 | Carotid extra left |
| 13 | Ophthalmic right |
| 14 | Ophthalmic left |
| 15 | Intradural carotid right |
| 16 | Intradural carotid left |
| 17 | MCA right distal |
| 18 | MCA left distal |
| 19 | Posterior cerebral right |
| 20 | Posterior cerebral left |

**A. Use of anatomical information in related works**

The authors in [1] used a vessel extractor tool [2] to only extract patches in the brain arteries. Similarly, the authors in [3] extracted the vessels from the MRA volumes using the method proposed in [4], although they do not specify in detail the reason for this vessel extraction. The authors in [5] performed a forced patch sampling to simulate the prevalence of aneurysms: specifically, they sampled 90% of patches in parts of the brain without aneurysms, and 10% of the patches in parts of the brain containing aneurysms. Instead, authors in [6] decided to perform a balanced patch sampling by extracting samples with a 50% of containing an aneurysm. The authors in [7] also tailored the patch sampling strategy to tackle class imbalance: specifically, they sampled the patches with aneurysms more often such that abnormal examples made up 30% of training iterations. [8] enhanced the vessel-to-background contrast of the DSA images before extracting training patches. [9] applied a region localization stage to only focus the analysis on the posterior communicating artery.

[1] Nakao et al., “Deep Neural Network-Based Computer-Assisted Detection of Cerebral Aneurysms in MR Angiography”

[2] Hanaoka et al., “HoTPiG: A novel geometrical feature for vessel morphometry and its application to cerebral aneurysm detection”

[3] Joo et al., “A deep learning algorithm may automate intracranial aneurysm detection on MR angiography with high diagnostic performance”

[4] Wang et al., “Threshold segmentation algorithm for automatic extraction of cerebral vessels from brain magnetic resonance angiography images”

[5] Sichtermann et al., “Deep Learning–Based Detection of Intracranial Aneurysms in 3D TOF-MRA"

[6] Shi et al., “A clinically applicable deep-learning model for detecting intracranial aneurysm in computed tomography angiography images”

[7] Park et al., “Deep Learning–Assisted Diagnosis of Cerebral Aneurysms Using the HeadXNet Model”

[8] Liu et al., “Deep neural network-based detection and segmentation of intracranial aneurysms on 3D rotational DSA”

[9] Duan et al., “Automatic detection on intracranial aneurysm from digital subtraction angiography with cascade convolutional neural networks”

**B. Vessel atlas registration**

We first registered the probabilistic vessel atlas to a structural anatomical scan of each patient (either T1- or T2-weighted) through a non-rigid registration (rigid + affine + symmetric normalization). Then, we registered the obtained warped volume to the TOF-MRA subject space through an affine registration.

**C. Weak label creation for 38 subjects with voxel-wise labels**

For the 38 subjects with voxel-wise labels, we created corresponding artificial weak labels. In other words, we converted the slice-by-slice annotations into spheres (we “*weakened”* the voxel-wise labels*).* The center of each artificial sphere corresponds to the center-of-mass of the corresponding voxel-wise label, while the diameter of the sphere corresponds to the maximum diameter of the voxel-wise label.

**D. Intensity criteria for negative patch sampling and sliding-window approach**

Both in the negative patch sampling and in the sliding-window approach, the patches need to fulfill 4 intensity criteria. In the negative patch sampling these intensity criteria serve to extract negative training patches which are comparable to the positive ones in terms of average intensity. Similarly, in the sliding-window approach, the criteria serve to retain only the candidate patches which have an average intensity comparable to the positive patches, thus discarding all the patches that do not contain vessels.

The following four criteria were chosen by looking at the intensities of positive patches (since we want to simulate their intensity):

1) the ratio $\frac{mean patch intensity}{\max\boldsymbol{patch} intensity}$ of the 3D TOF-MRA patch must be > 5^th^ percentile of the distribution of same ratios from positive patches (in-house + train ADAM). This condition ensures that the patch is locally bright enough.

2) the ratio $\frac{mean patch intensity}{\max\boldsymbol{volume} intensity}$ of the 3D TOF-MRA patch must be > 5^th^ percentile of the distribution of same ratios from positive patches (in-house + train ADAM). With *volume* we mean the whole TOF-MRA volume of the patient. This condition ensures that the patch is globally bright enough.

3) the ratio $\frac{mean patch intensity}{\max\boldsymbol{patch} intensity}$ of the 3D (co-registered) vessel atlas patch must be > 5^th^ percentile of the distribution of same ratios from positive patches (in-house + train ADAM). This condition ensures that the co-registered vessel atlas is non-empty for this patch, and thus the patch likely contains a vessel.

4) the ratio $\frac{mean patch intensity}{\max\boldsymbol{volume} intensity}$ of the 3D (co-registered) vessel atlas patch must be > 5^th^ percentile of the distribution of same ratios from positive patches (in-house + train ADAM). With *volume* we mean the whole vessel atlas co-registered to subject space. Again, this condition ensures that the patch is globally bright.

We always choose the conservative 5^th^ percentile of the distributions to ensure that the four conditions are extremely loose.

**E. Sliding-window approach**

Every test volume is explored with neighboring, overlapping patches. Each patch is fed to the trained network that outputs the corresponding semantic segmentation. The probabilistic segmentations are then binarized. Once the volume has been fully explored, all the binarized predictions are merged back to re-create the output volume. In this work, we used an overlap of 50% in all directions and we averaged overlapping predictions.

**F. Domain adaptation experiments between in-house and ADAM dataset**

Two experiments were performed to understand how knowledge could be transferred between the in-house and the ADAM dataset.

- *Model 8*: we only pre-trained the model on the ADAM dataset and then performed direct inference on the in-house dataset without finetuning. This experiment was carried out to see how features learnt on the ADAM dataset can generalize to the in-house dataset.
- *Model 9*: we only trained the model on the in-house dataset (i.e. without pre-train on ADAM). This experiment was performed to assess whether pre-training on ADAM helped or not for the final detection of aneurysms.

Here are the corresponding results:

| Model configuration | Anat-informed patch selection | Anat-informed sliding window | Labels of 38 added subs | Pre-train on ADAM | Train (finetune) on  in-house | Avg. Sensitivity (CI) | Avg. FP rate |
| --- | --- | --- | --- | --- | --- | --- | --- |
| *Model 8* | N.A. | N.A. | N.A. | Yes | No | 88/127 = 69% (60%, 75%) | 3.1 |
| *Model 9* | Yes | Yes | 38 voxel-wise | No | Yes | 100/127 = 79% (69%, 83%) | 1.0 |

Results of *Model 8* indicate that only pre-training on ADAM leads to lower performances on the test set of the in-house dataset. In fact, *Model 3* (pre-trained on ADAM, anatomically-informed patch sampling + anatomically-informed sliding window + 38 subjects with voxel-wise labels) statistically outperforms *Model 8* (two-sided Wilcoxon signed-rank test on the areas under the FROC curves, W = 845.0, p =0.01).

Instead, results of *Model 9* show that pre-training on ADAM actually does not improve performances significantly (two-sided Wilcoxon signed-rank test on the areas under the FROC curves, W = 767.0, p =0.9 when comparing *Model 3* and *Model 9*). As mentioned in the Discussion section of the manuscript, this finding suggests that further analyses are required to better exploit the pretraining on ADAM.


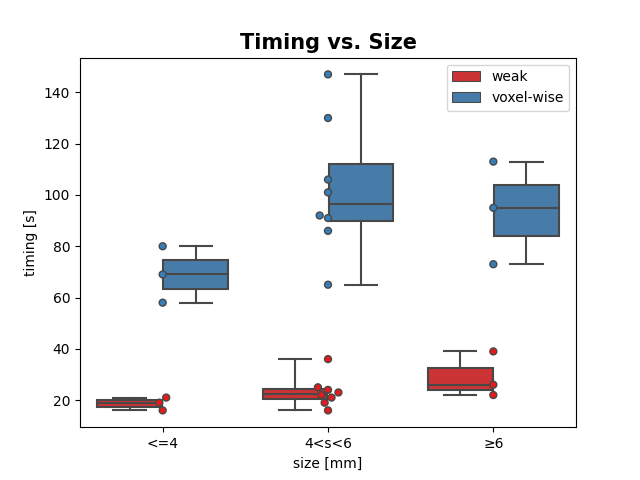


**Supplementary Fig 1**. Time needed to create the weak labels (red) and the voxel-wise labels (blue) with respect to aneurysm size. The timings are computed for 14 patients that were randomly sampled from the in-house cohort.


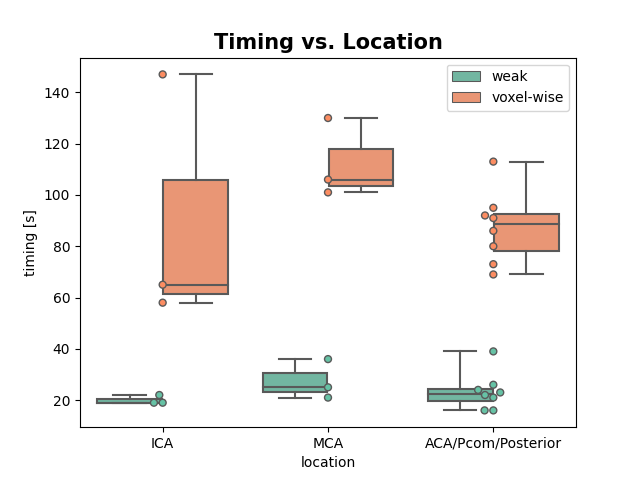


**Supplementary Fig 2**. Time needed to create the weak labels (green) and the voxel-wise labels (orange) with respect to aneurysm location. The timings are computed for 14 patients that were randomly sampled from the in-house cohort. ICA = Internal Carotid Artery, MCA = Middle Cerebral Artery, ACA = Anterior Cerebral Arteries, Pcom = Posterior communicating artery, Posterior = posterior circulation.


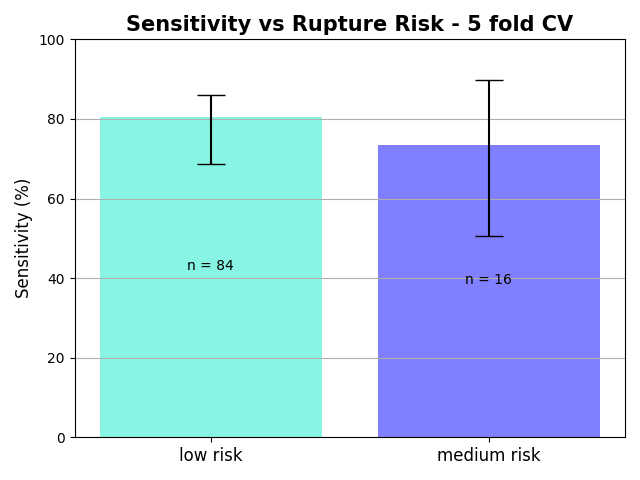


**Supplementary Fig 3**. Sensitivity of our anatomically-informed network (*Model 3,* Table 4) across the test folds with respect to the two risk-of-rupture groups. The *low-risk* group indicates aneurysms that will be monitored through imaging, but do not require any intervention. The *medium-risk* group includes more dangerous aneurysms that can be considered for treatment. Bar plots indicate the mean sensitivity value; error bars represent the 95% Wilson score interval. CV = cross-validation. n = number of sensitivity values in the distribution.


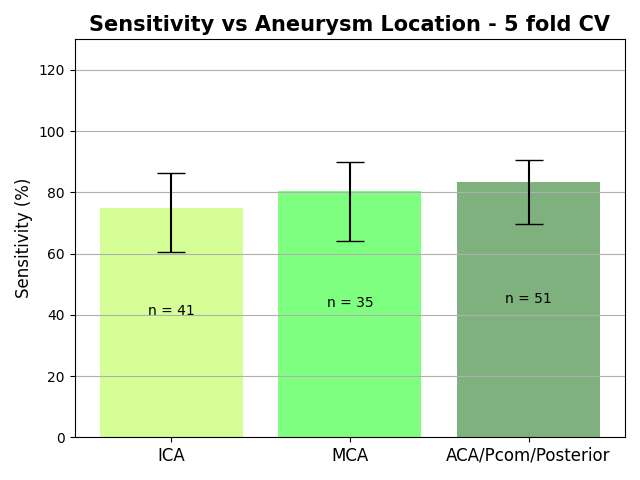


**Supplementary Fig 4**. Sensitivity of our anatomically-informed network (*Model 3,* Table 4) across the test folds with respect to the PHASES score aneurysm locations. ICA = Internal Carotid Artery, MCA = Middle Cerebral Artery, ACA = Anterior Cerebral Arteries, Pcom = Posterior communicating artery, Posterior = posterior circulation. Bar plots indicate the mean sensitivity value; error bars represent the 95% Wilson score interval. CV = cross-validation. n = number of sensitivity values in the distribution.


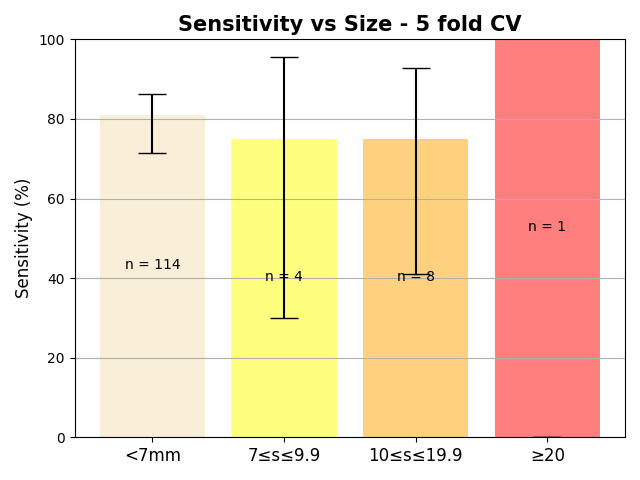


**Supplementary Fig 5**. Sensitivity of our anatomically-informed network (*Model 3,* Table 4) across the test folds with respect to the PHASES score sizes in mm. Bar plots indicate the mean sensitivity value; error bars represent the 95% Wilson score interval. CV = cross-validation. n = number of sensitivity values in the distribution.


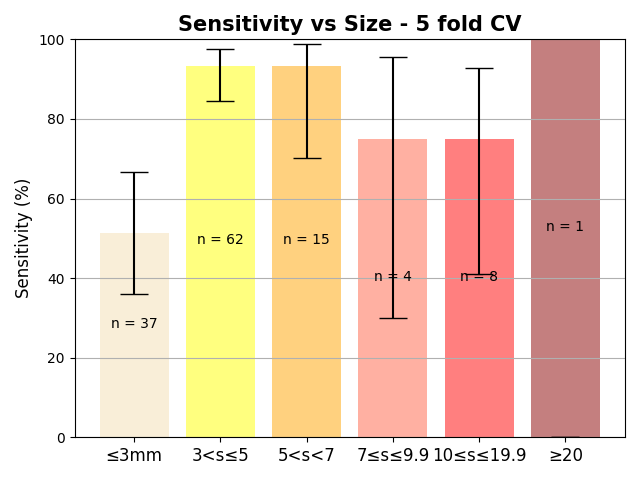


**Supplementary Fig 6**. Sensitivity of our anatomically-informed network (*Model 3,* Table 4) across the test folds with respect to a finer division of sizes. Even though the grouping in Supplementary Figure 5 (the one following the PHASES score sizes) is more clinically-relevant, a finer grouping is shown here since it is relevant to understand how the model performs with small aneurysms. Bar plots indicate the mean sensitivity value; error bars represent the 95% Wilson score interval. CV = cross-validation. n = number of sensitivity values in the distribution.
